# SS1P Immunotoxin Induces Markers of Immunogenic Cell Death and Enhances the Effect of the CTLA-4 Blockade in AE17M Mouse Mesothelioma Tumors

**DOI:** 10.3390/toxins10110470

**Published:** 2018-11-14

**Authors:** Yasmin Leshem, Emily M. King, Ronit Mazor, Yoram Reiter, Ira Pastan

**Affiliations:** 1 Laboratory of Molecular Biology, Center for Cancer Research, National Cancer Institute, National Institutes of Health, Bethesda, MD 20892, USA; Jasmin.leshem@gmail.com (Y.L.); emily.king.507@gmail.com (E.M.K.); mazorr@Medimmune.com (R.M.); 2 Laboratory of Molecular Immunology, Faculty of Biology and Technion Integrated Cancer Center, Technion-Israel Institute of Technology, Haifa 3200003, Israel; reiter@technion.ac.il

**Keywords:** immunotoxins, SS1P, anti-CTLA-4, mesothelioma, mesothelin, immunogenic cell death, immunotherapy, ATP, Calreticulin

## Abstract

SS1P is an anti-mesothelin immunotoxin composed of a targeting antibody fragment genetically fused to a truncated fragment of Pseudomonas exotoxin A. Delayed responses reported in mesothelioma patients receiving SS1P suggest that anti-tumor immunity is induced. The goal of this study is to evaluate if SS1P therapy renders mesothelioma tumors more sensitive to cytotoxic T-lymphocyte-associated antigen 4 (CTLA-4) immune checkpoint blockade. We evaluated the ability of SS1P to induce adenosine triphosphate (ATP) secretion and calreticulin expression on the surface of AE17M mouse mesothelioma cells. Both properties are associated with immunogenic cell death. Furthermore, we treated these tumors with intra-tumoral SS1P and systemic CTLA-4. We found that SS1P increased the release of ATP from AE17M cells in a dose and time-dependent manner. In addition, SS1P induced calreticulin expression on the surface of AE17M cells. These results suggest that SS1P promotes immunogenic cell death and could sensitize tumors to anti-CTLA-4 based therapy. In mouse studies, we found that the combination of anti-CTLA-4 with intra-tumoral SS1P induced complete regressions in most mice and provided a statistically significant survival benefit compared to monotherapy. The surviving mice were protected from tumor re-challenge, indicating the development of anti-tumor immunity. These findings support the use of intra-tumoral SS1P in combination with anti-CTLA-4.

## 1. Introduction

The immune system can recognize cancer-associated antigens and eliminate some cancer cells [1,2]. However, cancer cells that evolved into established tumors have outgrown or evaded the immune system. Immunotherapy is designed to shift the cancer-immune balance against tumors and promote tumor regression in patients [3]. One target being explored for immunotherapy is cytotoxic T-lymphocyte-associated antigen 4 (CTLA-4). It is an immune regulatory protein that is expressed on activated T cells and inhibits immune activation. An antibody that antagonizes CTLA-4 was effective in melanoma patients [4]. However, its use in other cancers has not yielded positive results so far [5]. An effort to find combination strategies with anti-CTLA-4 is warranted.

Immunogenic cell death promotes the recognition of dying cell antigens by the immune system. In the past, apoptotic cell death was considered tolerogenic; however, work done in mice shows that some neoplastic agents that kill cells by apoptosis can induce anti-tumor immunity [6,7]. Several markers of immunogenic cell death have been described, including the translocation of calreticulin from the endoplasmic reticulum (ER) to the cell surface and extracellular secretion of adenosine triphosphate (ATP). The induction of markers of immunogenic cell death can predict the ability of drugs to induce anti-tumor immunity [8], and these types of drugs would be good candidates for the potential synergy with anti-CTLA-4.

Immunotoxins are anti-cancer agents composed of a targeting domain genetically fused to a toxin [9]. In our lab, we develop immunotoxins that use truncated Pseudomonas exotoxin A (PE) as their payload fused to an antibody fragment that targets a tumor antigen. PE is one of the virulent factors of the gram-negative bacterium *Pseudomonas aeruginosa* [10]. After immunotoxins bind to cells and are internalized, the toxin is cleaved from the antibody domain, inhibits protein synthesis, and leads to cell death by apoptosis [11].

Mesothelin is a 40 kDa protein that under normal conditions is only expressed on mesothelial cells lining the pleura, peritoneal, and pericardium. The role of mesothelin in mice or humans is not known and mesothelin knocks out mice that do not have a noticeable phenotype [12]. Importantly, its expression is increased in many epithelial cancers including the lungs, pancreas, ovary, stomach, colon, and mesothelioma [13,14]. Mesothelin is being explored as a target for various immunotherapies [15].

SS1P is the first generation of anti-mesothelin immunotoxins which has been tested in patients with mesothelin-expressing cancers. As a single drug, it had only a modest effect. Out of 33 patients treated with bolus injections of SS1P, 4 had minor partial responses, and 19 had a stable disease [16]. One of the obstacles found in this study was the induction of rapidly evolving antibodies which neutralized the drug. To reduce the antibodies forming against SS1P, Hassan et al. combined SS1P with the immune modulating chemotherapies pentostatin and cyclophosphamide. In this study, 3 out of 10 patients had major regressions. All three continue to respond even when the drugs were discontinued and had major tumor regressions lasting up to 5 years [13,17]. We suspect that the direct cytotoxic effect of SS1P was accompanied by the induction of anti-tumor immunity.

LMB-100 (also known as RG7787) is a second generation anti-mesothelin PE immunotoxin. It contains a smaller fragment of PE (24 kDa) that is composed of enzymatically active domain III. In addition, it incorporates point mutations designed to reduce B cell recognition [18]. LMB-100 is currently being tested in clinical trials (NCT02798536, NCT03436732, NCT02810418).

Our previous work showed that injecting immunotoxins directly into tumors in combination with anti-CTLA-4 antibody had a synergistic anti-tumor effect in the 66C14 murine breast tumor model. Disease regressions were accompanied by long-term anti-tumor immunity indicated by the rejection of a second tumor challenge from the same cells [19].

In this study, we used a syngeneic AE17M murine mesothelioma tumor model to evaluate immunotoxin efficacy in the mesothelioma. AE17 cells were derived from the peritoneal cavity of C57BL/6 mice treated with asbestos and later modified to express human mesothelin (AE17M) [20,21]. We found that immunotoxin treatment promotes markers of immunogenic cell death in culture, and when injected directly into the tumors, immunotoxins enhance the effect of anti-CTLA-4 therapy.

## 2. Results

### 2.1. AE17 Cells Are Sensitive to Mesothelin Targeting Immunotoxins

**Figure 1:**
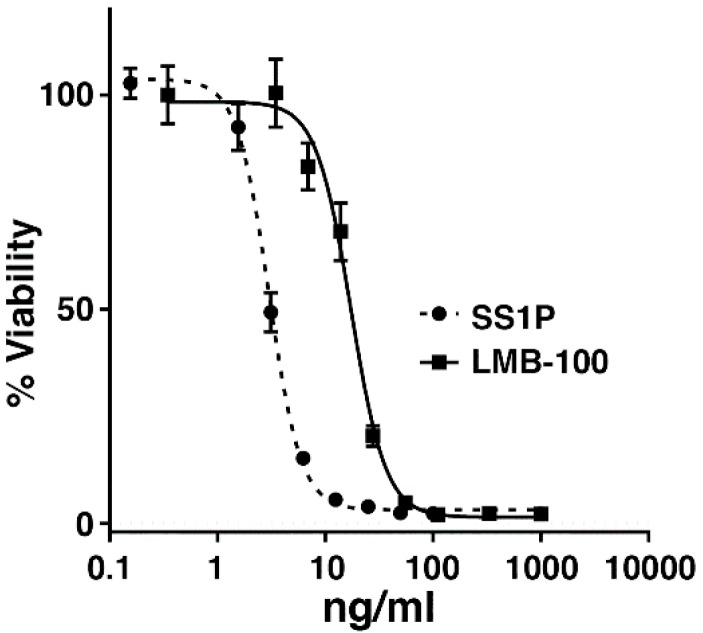
The cytotoxic activity of SS1P and LMB-100 in AE17 M cells. WST-8 cytotoxicity assays in AE17M cells after 3-day incubation with either SS1P or LMB-100.

To determine if murine mesothelioma AE17M cells are sensitive to anti-mesothelin immunotoxins in culture, the cells were cultured for three days with SS1P or LMB-100 at various concentrations and the cell viability was evaluated. We found that the cells were sensitive to both immunotoxins. SS1P was more cytotoxic than LMB-100. The half maximal inhibitory concentration (IC_50_) of SS1P was 3 ng/mL and that of LMB-100 was 17 ng/mL (Figure 1).

### 2.2. Anti-Mesothelin Immunotoxins Induce Extracellular Secretion of ATP

**Figure 2:**
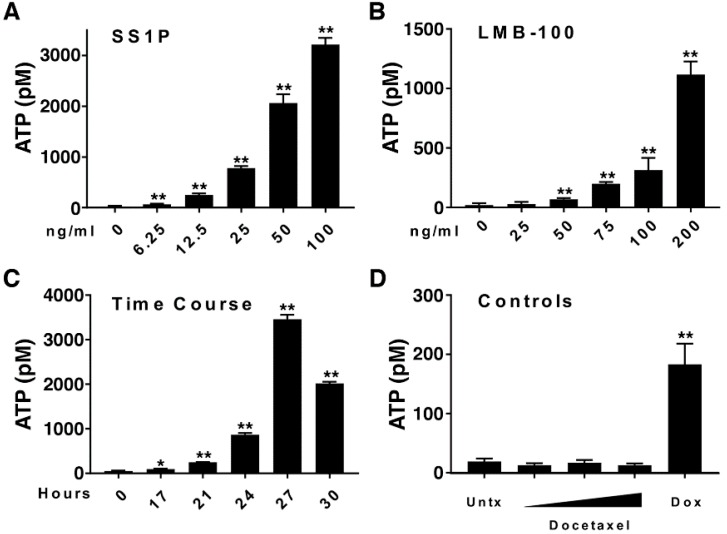
The anti-mesothelin immunotoxins promote the secretion of ATP. (**A**,**B**) Extracellular ATP in the media of AE17M cells after 24 h of incubation with either (**A**) SS1P or (**B**) LMB-100 at various concentrations. (**C**) Extracellular ATP in the media of AE17M cells incubated with 200 ng/mL LMB-100 at various time durations. (**D**) Extracellular ATP in the media in untreated AE17M cells or after 24 h of incubation with either 25, 250, or 2500 ng/mL of docetaxel or 13.4 µg/mL doxorubicin. * *p* < 0.05, ** *p* < 0.01. This experiment was repeated with similar results.

Extracellular ATP promotes dendritic cell activation and is considered a marker of immunogenic cell death [22]. We evaluated the ability of SS1P and LMB-100 to induce the secretion of ATP from dying AE17M cells. Figure 2A shows that the media of untreated AE17M cells contained 14 pM ATP and it significantly increased to 67, 795, and 3177 pM, when the cells were exposed to 6.25, 25, and 100 ng/mL of SS1P (*p* < 0.01), respectively. This indicates that SS1P induces ATP secretion and that the intensity of ATP secretion is dose-dependent. A similar trend was found when AE17M cells were incubated with various concentrations of LMB-100 (*p* < 0.01) (Figure 2B). The effect of immunotoxins on ATP secretion was also dependent on the duration of exposure. Extracellular ATP increased slightly from 43 pM in untreated cells to 87 pM after 17 h of exposure to LMB-100 and increased steeply to 3453 pM at 27 h (Figure 2C). A slight decrease in ATP was noted after 30 h of incubation with LMB-100, perhaps reflecting a decreased viability at this time point. This experiment was repeated twice with similar results.

We also evaluated the effect of docetaxel and doxorubicin on AE17M cells as negative and positive controls [8,23]. AE17M cells are sensitive to docetaxel with IC_50_ of 22 and 10.5 ng/mL after 24 and 72 h of incubation (Supplementary Figure S1). We treated AE17M cells with 25, 250, or 2500 ng/mL docetaxel and 13.4 µg/mL of doxorubicin and found that ATP was not secreted form AE17M cells treated with docetaxel but was secreted from cells that were exposed to doxorubicin (Figure 2D). Taken together, this data shows that anti-mesothelin immunotoxins induce the secretion of ATP in a similar manner to doxorubicin.

### 2.3. Anti-Mesothelin Immunotoxins Induce Surface Calreticulin

**Figure 3:**
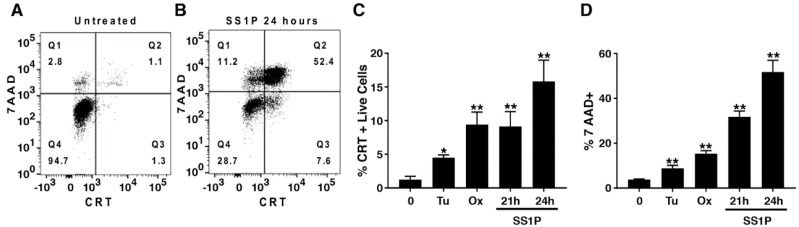
SS1P increases cell surface calreticulin. (**A**,**B**) Representative dot plots of (A) untreated AE17M cells or (B) cells exposed to 100 ng/mL SS1P for 24 h and stained with 7AAD and antibodies to calreticulin. (**C**) Percentage of calreticulin positive cells out of AE17M live cells (7AAD-) after exposure to 65 µM tunicamycin (24 h) or 5 µg/mL Oxaliplatin (24 h) or 100 ng/mL SS1P (21 or 24 h). (**D**) Percentage of 7AAD positive cells in the treatment conditions described in C. Pool data from two separate experiments is presented. CRT calreticulin, Tu Tunicamycin, Ox oxaliplatin, * *p* < 0.05, ** *p* < 0.01.

Calreticulin is another marker of immunogenic cell death. It facilitates the removal of dying cells and supports the efficient presentation of their antigens [24]. We explored the expression of surface calreticulin after exposure to SS1P. Figure 3A,B show representative histograms of untreated cells and cells exposed to SS1P. A distinct population of 7AAD negative cells that stained positive for calreticulin was present in quadrant 3 (Q3). In addition, we exposed AE17M cells to tunicamycin and oxaliplatin because those agents have been shown to cause lower and higher levels of calreticulin on other cell types [7,25]. For the final analysis, we excluded the dying cells and quantified the percentage of live cells that were positive to calreticulin.

Figure 3C shows that 1.2% of untreated cells are calreticulin positive. A significant increase in surface calreticulin was noted in all treatment conditions including treatment with tunicamycin, which increased the surface calreticulin to 4.3%, oxaliplatin, which increased it to 9.4%, and SS1P, which increased it to 9.1% after 21 h and to 15% after 24 h of incubation (*p* < 0.01). In all treatment conditions, the viability of the cells was decreased as determined by 7AAD staining. In particular, in cells that were treated with SS1P, the percentage of dead cells increased from 31% at 21 h to 51% at 24 h indicating that cells were actively dying (Figure 3D). These results indicate that SS1P causes calreticulin to be translocated to the surface of AE17M cells in a similar manner to oxaliplatin.

### 2.4. Direct Intra-Tumoral Injection of SS1P Inhibits Tumor Growth

**Figure 4:**
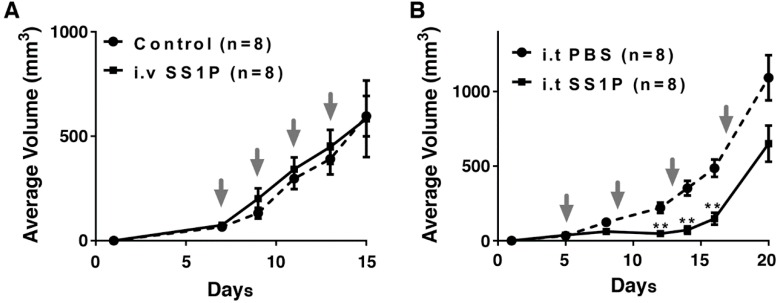
The in vivo anti-tumor effect of SS1P. (**A**) Average volume of AE17M tumors after receiving no treatment (control) or given intravenously SS1P (4 µg) every two days. (**B**) Average volume of AE17M tumors receiving direct intratumoral. SS1P (10 µg) or phosphate buffered saline (PBS) every four days. Treatment days are noted by the arrows. ** *p* < 0.01.

Because AE17M cells are more sensitive to SS1P compared to LMB-100, we evaluated the anti-tumor activity of SS1P in C57BL/6 mice. Cells were inoculated into mice and when tumors reached an average volume of 60 mm^3^, mice were treated intravenously (i.v.) with SS1P (4 µg) every two days for a total of four doses. Tumor volumes in SS1P treated mice were compared to tumors in untreated mice. We found that SS1P administered i.v. did not affect the growth rate of AE17M tumors (Figure 4A). We assumed that the drug concentration retained in the tumors is not high enough to kill tumor cells. To overcome this obstacle, we injected SS1P directly into the tumors. Figure 4B shows that mice treated with intra-tumoral (i.t.) SS1P (10 µg) every four days had a significant reduction in tumor size on days 12, 14, and 16, compared to mice injected with the vehicle alone (*p* < 0.01). However, the SS1P effect was temporary and the tumors grew rapidly after day 16 (Figure 4B).

### 2.5. Local SS1P Renders AE17M Tumors More Sensitive to Anti-CTLA-4 Effect

**Figure 5:**
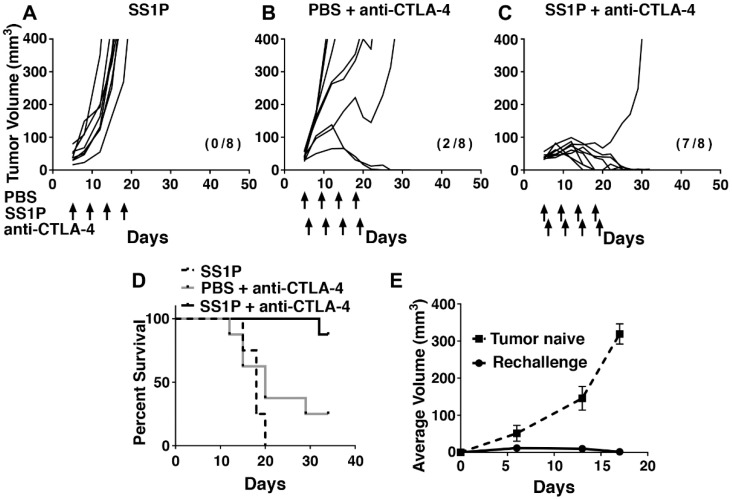
The SS1P and anti–CTLA-4 effect on AE17M tumor growth in mice. Individual tumor growth curves of AE17M tumors treated with (**A**) 8 µg SS1P i.t. alone, (**B**) PBS i.t. and 25 µg anti-CTLA-4 i.p., and (**C**) 8 µg SS1P i.t. and 25 µg anti-CTLA-4 i.p. (**D**) Long-term survival of mice described in (A–C). Survival of mice treated with SS1P and anti-CTLA-4 was significantly longer than that in the other groups (*p* < 0.05). (**E**) Mice that completely eradicate their tumor in (A–D) were re-challenged with additional AE17M cells implanted on the contralateral flank. The average tumor size is presented and compared to AE17M tumors growing on naïve mice (*n* = 5). The number of mice in complete regression and the total mice per group are shown in parentheses.

Based on the findings that SS1P enhances markers of immunogenic cell death, we hypothesize that SS1P will render tumors more sensitive to the activity of anti-CTLA-4. To test this hypothesis, we injected SS1P i.t. into AE17M tumors and treated with intraperitoneal (i.p.) anti-CTLA-4. AE17M tumor-bearing mice were treated with either i.t. SS1P (8 µg) or i.t. PBS every four days starting on day five when the average tumor volume was 40 mm^3^. Systemic anti-CTLA-4 (25 µg) was given i.p. every four days, starting on day six. Figure 5A shows that all AE17M tumors grew when SS1P was injected i.t. In mice treated with i.t. PBS and i.p. anti-CTLA-4, the tumor growth rate was decreased in 3/8 mice and lead to tumor eradication in two mice (Figure 5B). In mice treated with both anti-CTLA-4 and i.t. SS1P, tumor growth delay was noted in all mice and tumors were completely eradicated in seven out of the eight mice (Figure 5C). Figure 5D shows the survival of mice from various groups. The median survival of mice treated only with SS1P was 18 days and that of mice treated with PBS and anti-CTLA-4 was 20 days. A significant survival benefit was noted in mice treated with SS1P and anti-CTLA-4 compared to the control groups (*p* < 0.05). We repeated this experiment twice and found a significant survival benefit in both experiments (Supplementary Table S1).

Because LMB-100 is structurally distinct from SS1P, we evaluated its ability separately to enhance the effect of anti-CTLA-4. The trend of tumor control in mice was similar to that demonstrated with SS1P and anti-CTLA-4 and translated to a significant survival benefit (*p* < 0.05). However, the complete regression rate was slightly lower and ranged from 25% to 50%. A representative experiment and the survival data of mice in two additional experiments are shown in Supplementary Figure S2 and Supplementary Table S1.

### 2.6. Long-Term Anti-Tumor Immunity After SS1P and Anti-CTLA-4 Combination

To determine if anti-tumor immunity was induced by the combination treatment, we reinjected AE17M cells in the contralateral flank of mice that completely eradicated tumors (*n* = 9) and in tumor naïve mice (*n* = 5). Figure 5E shows that tumors were rejected by all of the cured mice, but grew well in tumor naïve mice, indicating that cured mice gained long-term anti-tumor immunity.

## 3. Discussion

In this study, we found that anti-mesothelin immunotoxins increased the expression of surface calreticulin and the extracellular secretion of ATP. Both are markers of immunogenic cell death. When SS1P is injected directly into AE17M tumors, it significantly enhances the effect of anti-CTLA-4 therapy. This result is in accordance with our previous report of a synergy between anti-CTLA-4 and locally injected anti-mesothelin immunotoxins found in the 66C14-M murine tumor model [19].

We found a significant increase in the number of cells that present calreticulin on their membrane and an increase in ATP secretion after exposure to cytotoxic levels of SS1P. Calreticulin is usually found in the ER but can translocate to the cell surface in response to ER stress and the phosphorylation of eukaryotic initiation factor 2 (eIF2). Once it reaches the cell surface, it acts as an “eat me” signal to phagocytic cells [26,27]. The ability of PE immunotoxins to induce ER stress and phosphorylation of eIF2 was previously shown in several cell lines [28,29]. PE’s mechanism of action is protein synthesis inhibition; thus, our result indicates that the surface calreticulin pathway can be activated without the production of new proteins.

We found that in response to SS1P or LMB-100, ATP is released from dying AE17M cells. Extracellular ATP is another marker of immunogenic cell death. It can bind to the P2X7 receptor on dendritic cells and prime them to promote anti-tumor immunity [22]. Much of the characterization of immunogenic cell death was done by vaccinating tumor-naïve mice with dying tumor cells that were pretreated in vitro with different chemotherapies [7,26]. The model of vaccination with dying cells is different from the clinical use of chemotherapies in which the therapy is given to cancer patients directly. Besides inducing immunogenic cell death in cancer cells, immunogenic chemotherapies, such as doxorubicin and oxaliplatin, are myelosuppressive and can induce leukopenia [30,31]. In patients, their ability to induce immunogenic cell death is countered by a decrease in circulating immune cells. Unlike chemotherapies, leukopenia is not one of the side effects of anti-mesothelin immunotoxins [16,32].

Our findings that localized injections to tumor site are a useful method of treatment has also been shown by Luther et al., who evaluated the use of the locally delivered 8H9scFv-PE38 immunotoxin into athymic rats bearing brain tumors. They found that locally delivered 8H9scFv-PE38 reduced the size of tumors, caused tumor necrosis and prolonged the survival of rats from 24 to 43 days [33].

Very few tumor models have been developed to evaluate the effect of PE immunotoxins on anti-tumor immunity. Ochiai et al. showed that when the MR1-1 immunotoxin was injected intratumorally immediately after inoculation of SMA560 EGFRvIII cells, it only prevented the formation of tumors in mice with an intact immune system. Moreover, the mice rejected a second challenge with the same cells, indicating that the MR1-1 immunotoxin mediates anti-tumor immunity [34]. Kawakami et al. showed that injecting the IL13-PE38 immunotoxin into D5 IL13α2 tumors slowed the growth rate of both injected and un-injected tumors growing in the same mice. This effect was abolished when the mice were depleted of CD4 and CD8 expressing cells, indicating that the effect depends on the adaptive immune system [35].

The AE17M mesothelioma tumor model is uniquely suitable to support clinical development of anti-mesothelin immunotoxins. Up to 80% of patients with mesothelioma have high mesothelin expression and thus are candidates for anti-mesothelin immunotoxin therapy [36]. In this model, injecting anti-mesothelin immunotoxins into the tumors had a transient anti-tumor effect and treatment with anti-CTLA-4 and i.t. PBS affected only a minority of tumors. However, the combination of i.t. anti-mesothelin immunotoxins with systemic anti-CTLA-4 induced tumor regression in the majority of the mice, supporting the idea that the combination of the two outperforms each treatment alone.

This work has a few limitations. One is that the AE17M tumor model did not respond to intravenous (i.v.) immunotoxins; thus, we were unable to evaluate whether i.v. immunotoxin sensitizes tumors to the therapeutic effect of anti-CTLA-4. Minor responses reported in clinical trials with i.v. SS1P suggests that i.v. immunotoxin can kill some tumor cells [16,32]. Nevertheless, the combination of locally injected immunotoxins with anti-CTLA-4 might be useful in unresectable local disease. As a single drug, TP-38 and NBI-3001 immunotoxins have occasionally resulted in the complete or durable partial responses in brain tumors [37,38,39,40]. The use of i.t. VB4-845 in cutaneous metastases resulted in the partial or complete regression in many of the injected tumors. Some patients also experienced regressions in un-injected tumor sites [41]. These effects might be further potentiated by a combination with anti-CTLA-4.

Altogether, this study shows that the local administration of anti-mesothelin immunotoxins potentiates the effect of anti-CTLA-4 in a murine mesothelioma model and induces markers of immunogenic cell death, providing preclinical support to pursue this combination strategy in the clinic.

## 4. Materials and Methods

### 4.1. Cell Culture and Reagents

SS1P was manufactured by ABL (Rockville, MD, USA). LMB-100 and anti-CTLA-4 (clone 9D9, isotype IgG2a) were manufactured by Roche. The AE17M cell line was kindly provided by Dr. Steve Albelda from the University of Pennsylvania. Cells were cultured in RPMI 1640 with 10% heat-inactivated FBS (incubated in a 56 °C water bath for 30 min) and supplemented with 100 U/mL penicillin, and 100 U/mL streptomycin. Cultures were maintained at 37 °C with 5% CO_2_.

### 4.2. Cytotoxicity Assays

Cells were plated at 2500 cells/well in 96-well flat-bottom plates and incubated overnight. Cells were treated with various concentrations of immunotoxins and incubated for 72 h. Cell viability was determined by a WST-8 cell counting kit (Dojindo Molecular Technologies, Inc, Kumamoto, Japan) per the manufacturer’s instructions. Absorbance was measured at 450 nm and normalized to 0% viability (cycloheximide treatment) and 100% viability (media).

### 4.3. Mouse Experiments

Female, wild-type C57BL/6 mice lot 027 at 6-9 weeks of age were purchased from Charles River. All mouse experiments followed NIH guidelines approved by the Animal Care and Use Committee of the National Cancer Institute (Animal Protocol LMB-014, date of approval: 3 March 2015). AE17M cells (2 × 10^6^) in PBS were inoculated subcutaneously on the flank. Tumor volumes were measured two to three times per week using a caliper. The tumor volume was calculated using the formula 0.4 × length × width^2^. Mice were euthanized if the tumor volume exceeded 400 mm^3^, if they became hypoactive, or lost more than 10% of their weight. The day of euthanasia was used to calculate survival. Immunotoxin was injected intravenously in a 100 µL volume, or directly into the tumor (i.t.) in a 30 µL volume. Prior to the i.t. injection, the injection site was sterilized with povidone-iodine and alcohol pads. Anti-CTLA-4 diluted with PBS was injected into the peritoneum (i.p.) at a volume of 200 µL. All mice were followed for 90 days or more. The cured mice were re-challenged with AE17M cells (2 × 10^6^) on the contralateral flank 40 days from the first tumor inoculation.

### 4.4. Flow Cytometry

AE17M cells were plated at 1 × 10^6^ cells in 10-cm tissue culture dishes and allowed to adhere overnight. Cells were treated with either 65 µM tunicamycin (Sigma, St. Louis, MO, USA), 5 µg/mL oxaliplatin (Teva) or 100 ng/mL SS1P. All cells were harvested (including cells floating in the media) and stained with a rabbit polyclonal anti-calreticulin antibody (ab2907, Abcam, Cambridge, UK) at 1:200 for 60 min. Secondary anti-Rabbit FITC antibody (ab6717, Abcam) was added at 1:1000 for 30 min. Dead cells were labeled using 7AAD (BD Pharmigen, San Diego, CA, USA) and excluded from the analysis. Data were acquired on a FACSCanto II flow cytometer (BD Bioscience, Franklin Lakes, NJ, USA) and analyzed using FlowJo.

### 4.5. ATP Assay

AE17M cells were plated at 8 × 10^4^ in a 24 well plate and allowed to adhere overnight. Cells were treated with either Doxorubicin (Pfizer, New York, NY, USA), Docetaxel (Winthrop, Bridgewater, NJ, USA) or anti-mesothelin immunotoxins in various concentrations and time durations. Next, the plates were spun at 1000 rpm for three minutes to reduce cell contamination and the supernatant from each well was transferred to a different 96 plate and ATP measured using the ENLITEN (Promega, Madison, WI, USA) kit according to the manufacturer’s instructions. Each 24-well plate was processed and evaluated separately to reduce the processing time until ATP was measured. Bioluminescence was analyzed using Victor^3^ (PerkinElmer, Waltham, MA, USA).

### 4.6. Statistical Analyses

Statistical analyses and graphing were performed with the GraphPad Prism software. The log-rank (Mantel-Cox) test was used to compare survival of mice. The Mann–Whitney test was used to compare the percentage of calreticulin positive cells and ATP values between groups. Error bars represent SEM.

## Supplementary Materials

The following are available online at http://www.mdpi.com/2072-6651/10/11/470/s1, Figure S1: Cytotoxic activity of Docetaxel in AE17 cells. Figure S2: LMB-100 and anti–CTLA-4 effect on AE17M tumor growth in mice. Table S1: Survival of mice treated with anti-mesothelin immunotoxins and anti-CTLA-4.
