# Supplementary material for: SS1P Immunotoxin Induces Markers of Immunogenic Cell Death and Enhances the Effect of the CTLA-4 Blockade in AE17M Mouse Mesothelioma Tumors
Source: Toxins (Basel). 2018 Nov 14;10(11):470. doi: 10.3390/toxins10110470 (PMC6265743; doi:10.3390/toxins10110470)
Supplement: Supplementary file 1 [file toxins-10-00470-s001.pdf]

Supplementary Materials: SS1P Immunotoxin Induces Markers of Immunogenic Cell Death and Enhances the Effect of CTLA-4 Blockade in AE17M Mouse Mesothelioma Tumors

Yasmin Leshem, Emily M King, Ronit Mazor, Yoram Reiter and Ira Pastan

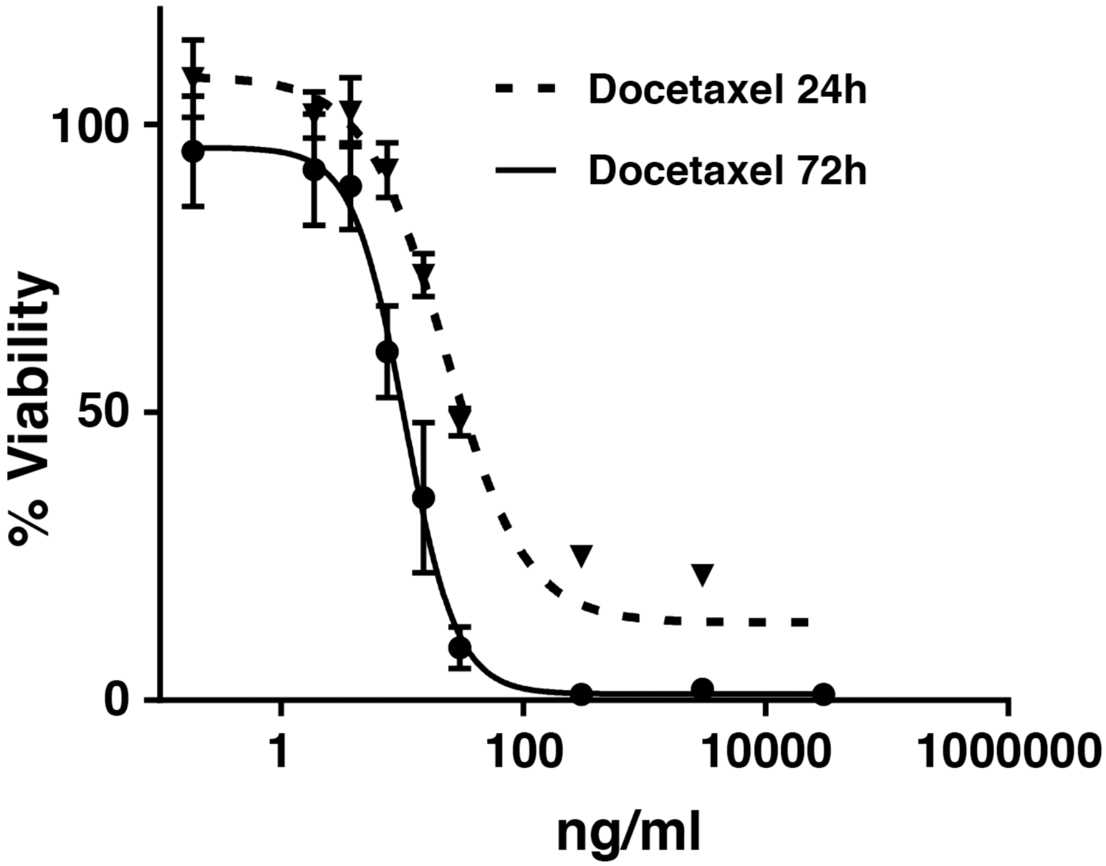

Figure S1. Cytotoxic activity of Docetaxel in AE17 cells. WST-8 cytotoxicity assays in AE17M cells after 24 h or 72 h of incubation with docetaxel.

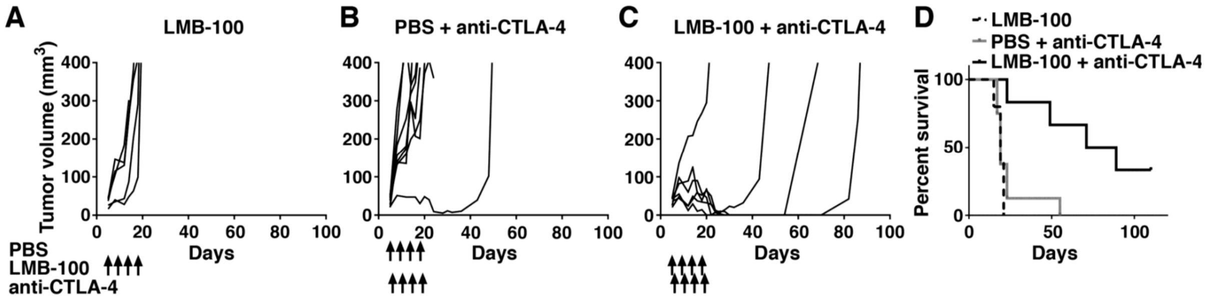

Figure S2. LMB-100 and anti–CTLA-4 effect on AE17M tumor growth in mice.(**A**–**C**) Individual tumor growth curves of AE17M tumors treated with (**A**) 30 μg LMB-100 i.t. alone (*n* = 5), (B) PBS i.t. and 25 μg anti-CTLA-4 i.p.(*n* = 8), and (**C**) 30 μg LMB-100 i.t. and 25 μg anti-CTLA-4 i.p.(*n* = 6). (**D**) Long-term survival of mice described in **A**–**C**. Survival of mice treated with LMB-100 and anti-CTLA-4 was significantly longer than that in the other groups ($p < 0.01$).

Group	Experiment	CR out of Total (*n*/*n*)	Median Survival
**SS1P + Anti-CTLA-4**	1	4/6	>90 *
	2	1/8	31 * [I]
	3	7/8	>90 *
**LMB-100 + Anti-CTLA-4**	2	2/6	73 *
	3	4/8	45 *
	4	2/8	37 *
**SS1P alone**	1	0/6	19
	2	0/8	21
	3	0/8	18
**LMB-100 alone**	2	0/5	19
	3	0/8	20
	4	0/8	22
**PBS + Anti-CTLA-4**	1	1/6	28
	2	0/8	19 [I]
	3	2/8	20
	4	0/5	19

Table S1. Survival of mice treated with anti-mesothelin immunotoxins and anti-CTLA-4. Survival of mice in four separate experiments numbered 1 to 4 in which AE17M tumor bearing mice were treated with either i.t. PBS or i.t. SS1P (8 to 10 μg) or i.t. LMB-100 (30 to 60 μg) with or without i.p. anti-CTLA-4 (25 μg). i.t. treatment was given on days 5, 9, 13 and 17 and anti-CTLA-4 on days 6, 10, 14 and 18. n, number of mice.CR, complete regression * a significant survival benefit was found compare to that of mice treated with PBS and anti-CTLA-4 in the same experiment ($p < 0.05$). [I] inexperienced operator was giving i.t. treatment.
